# Finally, a step forward in nomenclature of subchondral nonneoplastic bone lesions

**DOI:** 10.1186/s12891-020-3071-5

**Published:** 2020-01-20

**Authors:** William Fulton Postma, Evan Micheaelson, Laura Keeling

**Affiliations:** Department of Orthopaedic Surgery, Medstar Georgetown University Hospital, Washington DC, USA

**Keywords:** Bone lesions, Subchondral lesions, Bone edema, Subchondral cysts, Edema-like Lsions, Subchondral edema, Subchondral fracture, Osteochondral lesion, Bone bruise

## Abstract

This paper is a commentary on the article entitled “Nomenclature of Subchondral Nonneoplastic Bone.

Lesions1” by Gorbachova, Amber, Beckmann, Bennett, Chang, Davis, Gonzalez, Hansford, Howe, Lenchik, Winalski, and Bredella. The purpose of this commentary is to provide an orthopaedic perspective on the aforementioned article and critique their analysis and proposal regarding nomenclature of subchondral bone lesions. It provides an overview and a section by section evaluation of a well-designed and executed article.

The paper entitled “Nomenclature of Subchondral Nonneoplastic Bone Lesions [1]” by Gorbachova, Amber, Beckmann, Bennett, Chang, Davis, Gonzalez, Hansford, Howe, Lenchik, Winalski, and Bredella is a welcome addition and clarification to what is a muddied and confusing pool of terms used to describe subchondral pathologies. The proposed revised nomenclature has been well thought out on a clinical, histopathologic, and imaging basis, taking into account history of injury and chronicity when applying specific phrasing. As an orthopedic surgeon, the universal adoption of this nomenclature will not only help with communication of the underlying disease process, but will also aid in prognosis and treatment which an ideal classification does. In my opinion, as both radiologists and orthopedic surgeons, we too often loosely use terms such as “bone edema” and “bone bruises” without fully understanding the underlying pathologic processes and implications. This article represents an applauded step towards universalization of appropriate terminology and improved understanding.

Generally speaking, I understand the rationale and concur with the proposed terms put forth in this paper but there are some highlights from an orthopaedic standpoint. The discussion of “edemalike marrow signal” and the avoidance of the term “bone marrow lesion” in the situations where pathologies lack specificity. The term bone marrow lesions can cause confusion and undue anxiety on physicians at times, but also on patients reading their reports. One of the most important discussions in the paper centers around “osteochondral lesions” and the importance of clarification when possible rather than a waste basket usage of this term. This becomes especially important in the setting of osteochondral defects and osteochondritis dissecans as proper evaluation and terminology of the pathology helps to drive decision-making and ultimately surgical vs non-surgical treatments. This is also true of epiphyseal or cartilage collapse as improper use has the potential for patient harm. For instance, as pointed out in the discussion in the paper, once articular collapse has occurred the typical disease process resulting in that collapse is non-reversible and most oftentimes progressive. Therefore, if articular collapse is used improperly a patient may undergo unnecessary surgical intervention. The same can also be said for osteonecrosis and transient osteoporosis of the hip as well as the other categories but there is no need to belabor the point as the authors do a nice job laying it out in the paper itself. Here is a brief commentary from an orthopaedic perspective presenting the material as they did in the original paper. Please refer to that paper for the full description and reasoning for normalizing terminology.

## Edema like marrow signal intensity

The discussion surrounding this terminology is especially important when distinguishing between an acute (ACL injury pattern) versus a chronic condition (various arthritides). All too often bone marrow edema is used improperly and universally and I agree with its avoidance.

## Cystlike lesion

The authors, again, pointed out the misuse of the term cyst and bone marrow lesions that do not accurately describe the pathology process occurring and may cause further confusion. The term cystlike lesion or changes dampens that confusion especially when describing areas related to arthritis that have many times been described as bone marrow lesions. From a practical clinical standpoint, the term “bone marrow lesion” oftentimes results in patient trepidation as they read their report and then google that phrase. This exact scenario occurred in my clinic a couple of weeks ago as the patient believed they had a malignant process.

## Osteochondral lesion

This term should be avoided unless it cannot be more accurately described, which is rare.

## Osteochondral defect

The paper does an excellent job describing when this term should be used. I wholeheartedly agree with avoiding the abbreviation “OCD” for these lesions as it implies osteochondritis dissecans, which may or may not be the underlying cause of the defect. Using OCD to describe all of these is quite confusing to physicians and students. OCD should only be used to describe the distinct entity of osteochondritis dissecans.

## Osteochondral fracture and Subchondral fracture

Osteochondral fracture involves the subchondral bone extending to the cartilage surface whereas subchondral fracture involves only the subchondral bone without extension to the surface. In both scenarios a fracture line or contour deformity needs to be present in order to classify it as such. This is put forth in an organized fashion in the paper. My addition to this is the avoidance of these terms in the setting of signal change in these areas with no fracture line or contour deformity as many times these are overcalled. One particular scenario is seen with ACL injuries where it is more of a “bone contusion” although fractures do occur.

## Epiphyseal collapse

This particular term has many implications especially in the setting of avascular necrosis (AVN) as it signifies irreversible damage and also implies further progression and collapse will occur. It is also reserved for atraumatic processes as pointed out by the authors. It is synonymous with articular collapse which is acceptable as well.

## Acute traumatic Osteochondral lesion

This is not synonymous with acute chondral lesion as an osteochondral lesion implies bony involvement and completely changes the treatment options. This differentiation is especially important.

## Subarticular stress response

Subarticular stress response and stress reaction can be used interchangeably in my opinion. This can be a prequel to a stress fractures but the term stress fracture should not be used unless a fracture line is present. These are chronic repetitive injury patterns, not acute.

## Osteochondritis Dissecans

Osteochondritis dissecans is a distinct entity and is differentiated from osteochondral defect. The usage of OCD should be reserved for osteochondritis dissecans and is not interchangeable for an osteochondral defect. The authors again describe this well.

## Subchondral insufficiency fracture (SIF)

The authors’ discussion of this entity leaves little to be added. I would like to highlight that the clinical history is especially important when using this term. If possible, further description should be added to better ascertain the underlying disease process.

## Spontaneous osteonecrosis of the knee (SONK)

This term has become quite controversial as many believe this is not a distinct entity but rather results from other entities. One specific entity that has garnered much attention recently is meniscal root tears. Therefore, I agree with avoidance of this term altogether and replacement with subchondral insufficiency fracture (SIF).

## Osteonecrosis

The distinction between this entity and SIF should be made whenever possible. When describing osteonecrosis, from an orthopaedic perspective, the most important aspect is whether there has been epiphyseal collapse as this guides treatment. As pointed out above and in the paper, once epiphyseal collapse has occurred the disease is progressive regardless of treatment. Other than the diagnosis of osteonecrosis, the presence or absence of epiphyseal collapse should be described.

## Rapidly destructive osteoarthritis and osteoarthritis

In general, in orthopaedics there is not much of a need to distinguish between rapidly destructive osteoarthritis and osteoarthritis. However, there is a need to distinguish between milder levels of osteoarthritis and moderate to severe levels as this comes into play with procedural options for patients. This is especially true in the setting of hip impingement as patients with mild OA in the setting of impingement can still do well with arthroscopy while arthroscopy has no role once hip impingement has progressed to moderate to severe. In the hip it would be helpful to use the Tonnis classification as grade 2 and beyond are not recommended for arthroscopic treatment. While most surgeons understand this and do not need this delineation as they can recognize it, insurance companies are now taking this into account when deciding approval for surgeries.

In closing, the authors should be congratulated for their work and attempt to step away from historical nomenclature that misrepresents pathologic processes. We need to follow suit in other areas of medicine as our understanding of different disease entities deepens.

## Data Availability

N/A
